# Mevalonic Aciduria in a Pediatric Patient: A Case Report and Literature Review of Neuroimaging Findings

**DOI:** 10.7759/cureus.65209

**Published:** 2024-07-23

**Authors:** Mateus A Esmeraldo, Izaely R Prates, Leandro T Lucato, Alcino A Barbosa Junior

**Affiliations:** 1 InRad - Institute of Radiology, Clinics Hospital of the Medical School of the University of São Paulo, São Paulo, BRA

**Keywords:** metabolic disorders, hyperimmunoglobulin d syndrome, mvk gene mutation, neuroimaging, mevalonic aciduria

## Abstract

Mevalonic aciduria is a rare autosomal recessive disorder resulting from mevalonate kinase deficiency. Neuroimaging findings associated with the disease have been documented in only a few case reports. We present a case of mevalonic aciduria with both already reported and novel neuroimaging findings and conduct a literature review regarding the role of neuroimaging in the understanding and diagnosis of mevalonate kinase deficiency disorders. The brain magnetic resonance imaging of the reported case revealed several notable findings, including polymicrogyric cortical thickening, an interhypothalamic adhesion or small hypothalamic hamartoma (findings not classically associated with mevalonic aciduria), and mild cerebellar atrophy. This case underscores the significance of recognizing the diverse spectrum of neuroimaging findings associated with the disease, encompassing both well-documented features and those that have not been traditionally reported.

## Introduction

Mevalonic aciduria (MA), the most severe form of mevalonate kinase deficiency, is an autosomal recessive disorder resulting from the deficiency of mevalonate kinase, an enzyme of the cholesterol and isoprenoid biosynthetic pathway. The disorder encompasses a spectrum, ranging from a mild form known as hyperimmunoglobulinemia D and periodic fever syndrome to the more severe manifestation as MA. A genetic connection was established between the two clinically described phenotypes through the identification of a shared genetic anomaly in the *MVK* gene on chromosome 12q24.3 that triggers episodes of hyperinflammation marked by elevated interleukin 1β (IL-1β) secretion [1].

The epidemiology of mevalonate kinase deficiency is largely uncertain, with regional variations and the highest prevalence in the Netherlands. The Eurofever registry suggests a global impact on at least 300 people, predominantly with the hyperimmunoglobulinemia D and periodic fever syndrome phenotype. Its distribution in the United States remains unclear, with the largest study involving just 20 patients [1].

Since its initial description in 1985 by Berger et al., the clinical picture of this condition has expanded significantly [2]. It is characterized by a constellation of symptoms, such as autoinflammatory flares causing fever, abdominal pain, mucoid and cutaneous lesions, and arthralgias. Severe cases, such as MA, may exhibit developmental delay, physical dysmorphisms, psychomotor disability, ataxia, ocular abnormalities, and hepatosplenomegaly [1,3].

## Case presentation

We document the case of a four-year-old male child, who was delivered full term via cesarean section following an uneventful gestational period. The neonatal period, however, was complicated by a 24-day hospital admission immediately postpartum due to complications arising from meconium aspiration, concurrent infection, and anemia.

The patient’s clinical history during the first two years of life was marked by recurrent infectious diseases, including pneumonia, pharyngitis, otitis media, and gastroenteritis, along with the finding of hepatosplenomegaly. Therefore, an exhaustive diagnostic investigation became imperative.

In advance of a definitive diagnosis, a systematic management plan was enacted, incorporating the utilization of corticosteroids during periods of symptomatic manifestation. Additionally, a therapeutic course of canakinumab was tested for three years. In the absence of anti-IL1 treatment, the patient was observed to endure multiple symptomatic periods, typified by fever and infections, yet these periods demonstrated a discernible amelioration following corticosteroid intervention.

A subsequent genetic panel analysis identified a mutation in the *MVK* gene, thereby confirming the diagnosis of a disorder associated with mevalonate kinase deficiency.

Neuroimaging findings

**Figure 1:**
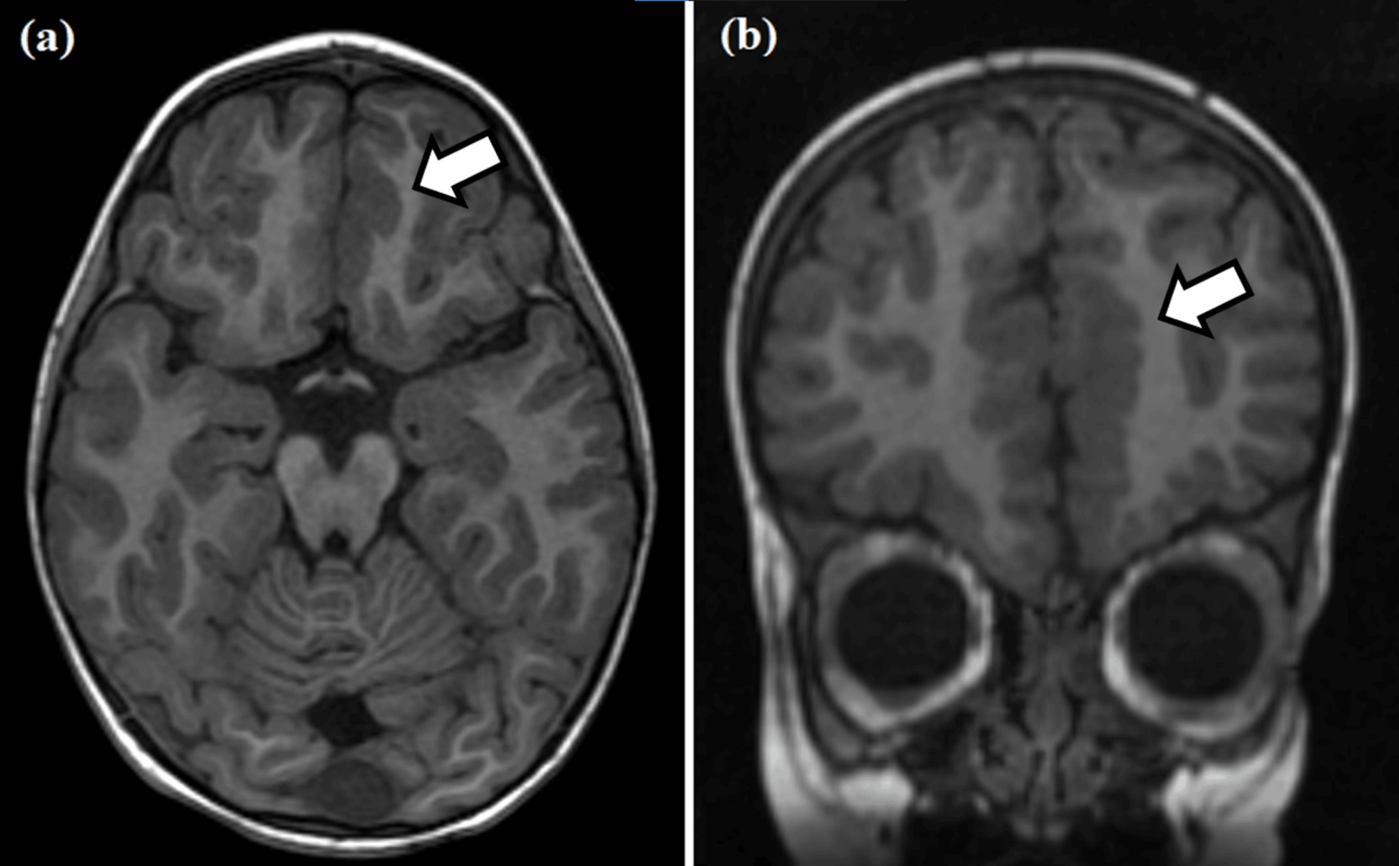
Axial (a) and coronal (b) T1-weighted MRIs. Cortical thickening and micro-lobulation at the cortico-subcortical interface (white arrows) are noted in the mesial surface of the left frontal pole, specifically at the transition from the superior frontal gyrus to the gyrus rectus.

Slight cortical thickening and micro-lobulation at the cortico-subcortical interface were noted in the mesial surface of the left frontal pole, specifically at the transition from the superior frontal gyrus to the gyrus rectus (Figure 1). A slight thinning was detected at the transition of the posterior portion of the corpus callosum trunk.

**Figure 2:**
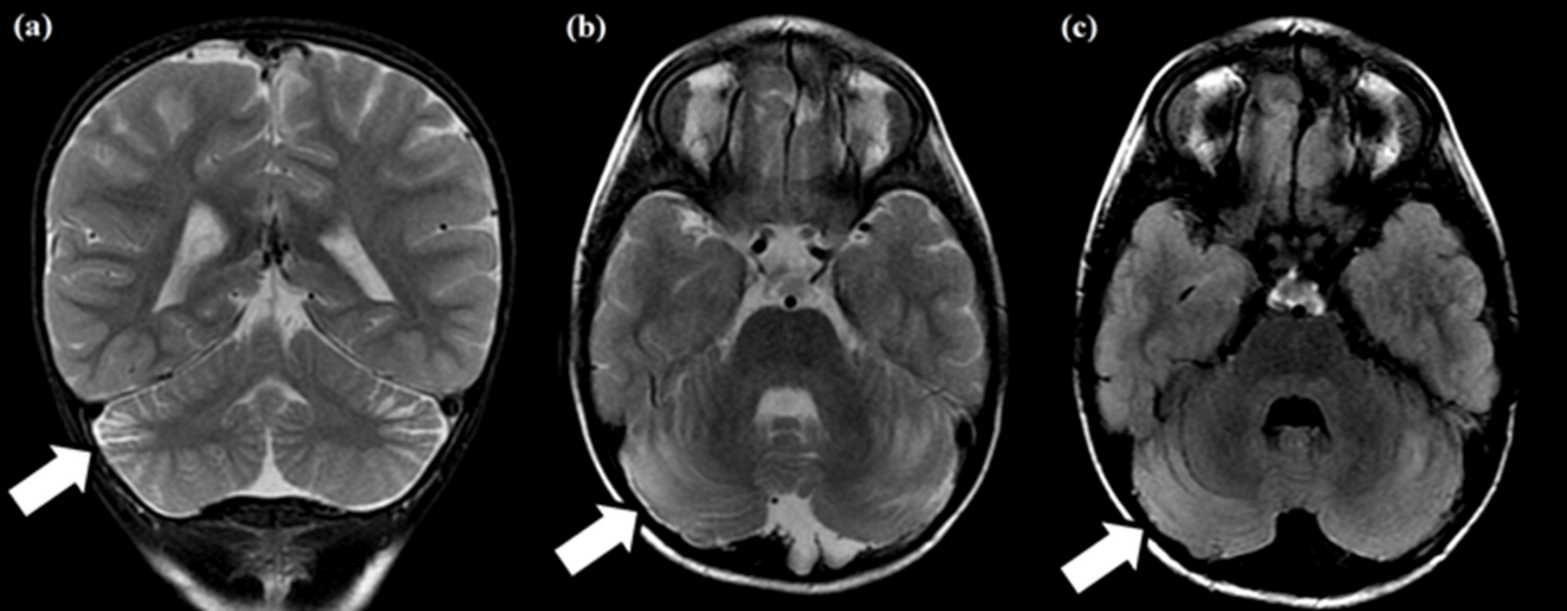
Coronal T2-weighted (a), axial T2-weighted (b), and fluid-attenuated inversion recovery (c) MRIs. Cortico-subcortical bands with hyperintensities are noted in the periphery of the middle third of the cerebellar hemispheres, associated with slight regional volumetric reduction (white arrows). These findings likely indicate regional gliosis with slight atrophy.

Cortico-subcortical bands with T2/fluid-attenuated inversion recovery (FLAIR) hyperintensities were found in the periphery of the middle third of the cerebellar hemispheres. These presented no diffusion restriction or post-contrast enhancement and were associated with focal widening of regional sulci and fissures (slight regional volumetric reduction). These findings likely indicated regional gliosis with slight atrophy (Figure 2).

**Figure 3:**
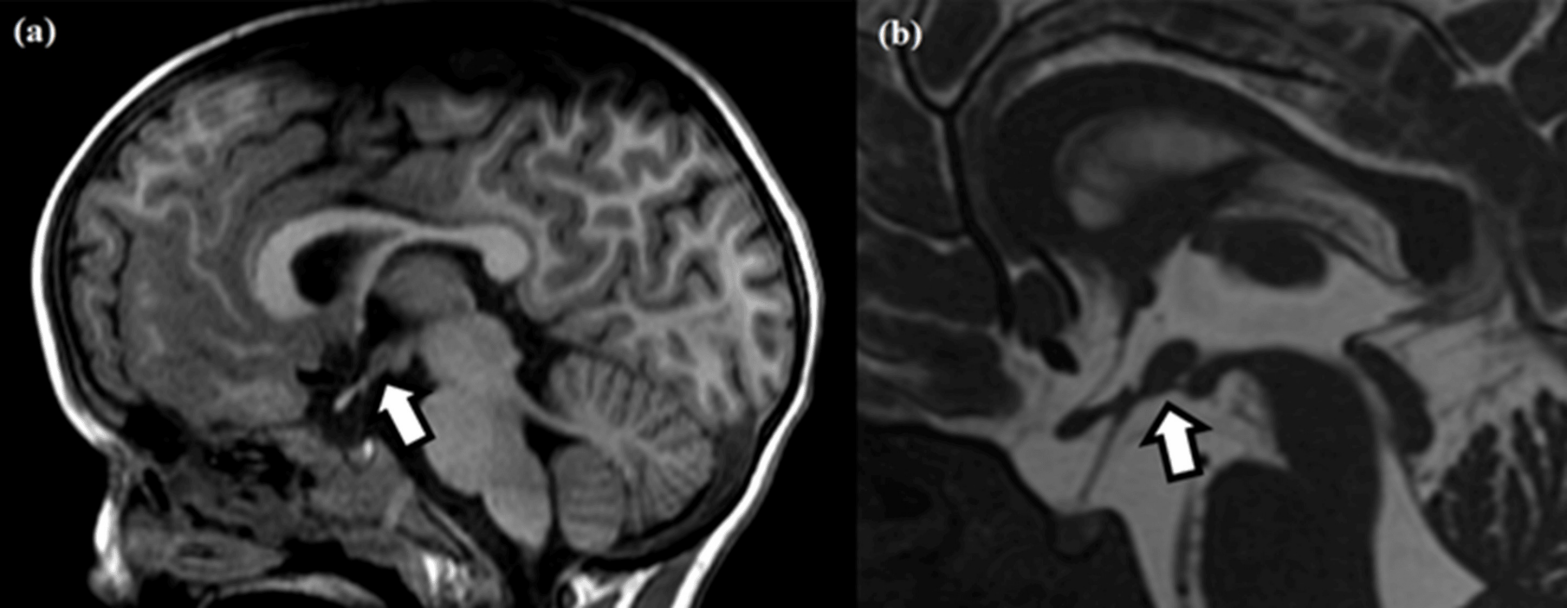
T1-weighted (a) and fast imaging employing steady-state acquisition (b) sagittal MRIs. Slight thinning of the corpus callosum isthmus and an oval formation with signal intensity similar to gray matter noted in the tuber cinereum, connecting both sides of the hypothalamus (white arrows).

An oval formation with a similar signal to the gray matter was noted in the tuber cinereum, communicating with the two sides of the hypothalamus, with no diffusion restriction or post-contrast enhancement, which could either represent an interhypothalamic adhesion or a small hypothalamic hamartoma (Figure 3). The proton spectroscopy study did not reveal significant alterations in the relationship between the metabolites or any abnormal peaks.

## Discussion

**Figure 4:**
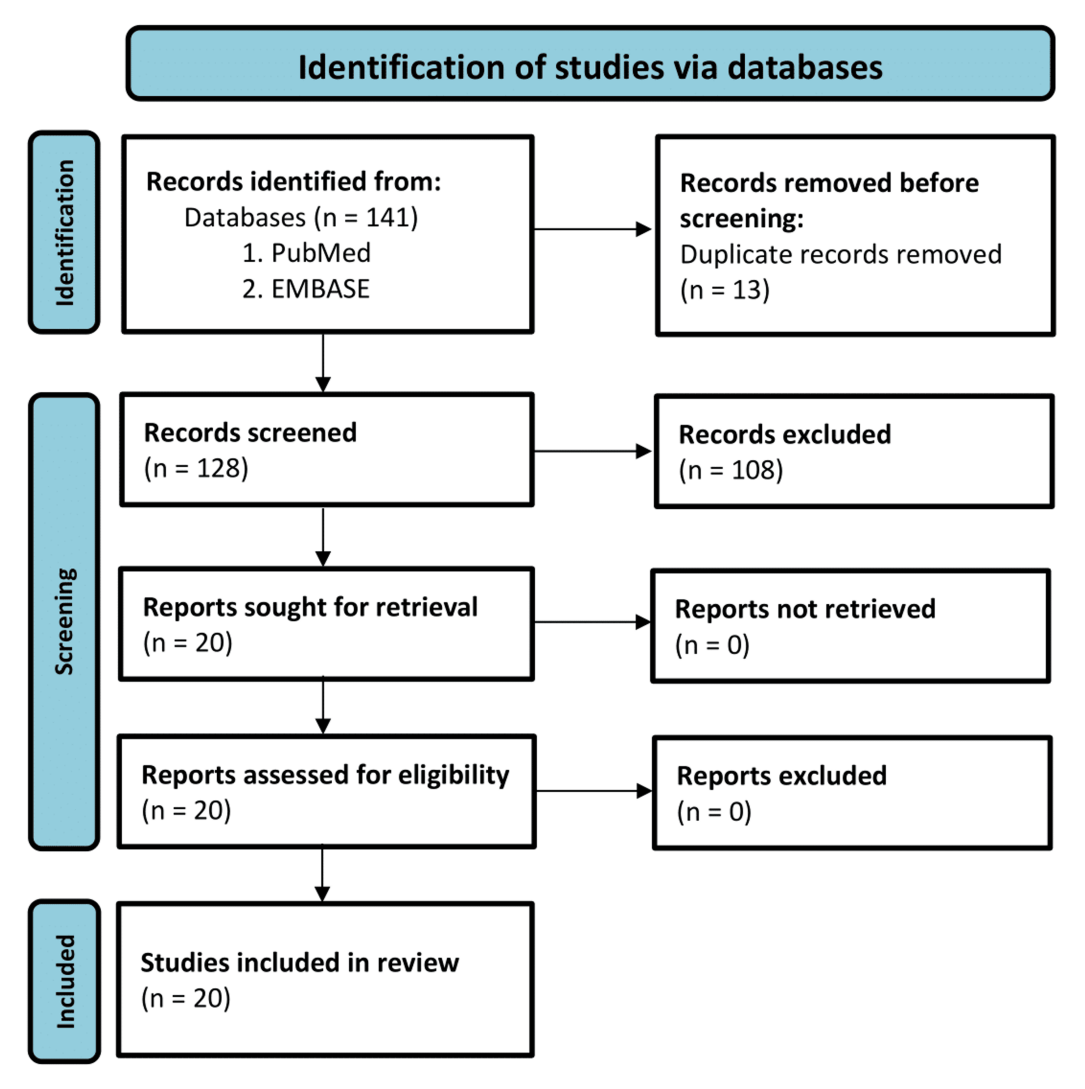
Flow diagram of the systematic review.

To gather relevant articles, a comprehensive search was conducted following the Preferred Reporting Items for Systematic Reviews and Meta-Analyses 2020 statement guidelines to ensure transparency, completeness, and reproducibility (Figure 4). The following specific keywords were used: (“mevalonic aciduria” OR “mevalonate kinase deficiency” OR “hyperimmunoglobulinaemia d”) AND (“magnetic resonance imaging” OR “MRI” OR “computed tomography” OR “imaging” OR “neuroimaging” OR “spectroscopy.”) The search was performed independently in PubMed and EMBASE databases. The last search was performed on May 12, 2024. A total of 18 articles were retrieved from PubMed and 123 articles from EMBASE.

After removing duplicates, 128 articles remained for further evaluation. The inclusion criteria consisted of articles that provided information on neuroimaging findings related to mevalonate kinase deficiency. The exclusion criteria were articles that did not focus directly on the disease or lacked specific neurological imaging data. Based on these criteria, 108 articles were excluded, leaving 20 articles for the final analysis and retrieval of their full-text versions.

The 20 selected articles were carefully reviewed to extract relevant information about the neuroimaging findings in mevalonate kinase deficiency disorders. Findings from these articles are compiled and synthesized in Table 1. The prevalence of neuroimaging findings in MA is challenging to determine as they are primarily described in case reports. However, it is essential to note that, based on the case series, severe neurological symptoms are only observed in a minority of patients. A registry analysis from Eurofever, which included 114 cases of mevalonate kinase deficiency, reported cerebellar syndrome in two patients (1.7%) and intellectual disability in two others (1.7%), with headache being the most common neurological symptom, occurring in 43 patients (37.7%) [4].

**Table 1:** Summary of relevant information and neuroimaging findings of the selected articles in the literature review. MRI: magnetic resonance imaging; FLAIR: fluid-attenuated inversion recovery

Article	Images	Article type	Relevant information	Brain MRI	Ultrasound	Optical coherence tomography	Photo
Kozich et al [5]	No	Case report	Brain imaging methods revealed agenesis of vermis cerebelli and cerebellar atrophy	Agenesis of vermis cerebelli and cerebellar atrophy	-	-	-
Hoffmann et al. [6]	No	Case series	Neuroimaging studies revealed selective and progressive atrophy of the cerebellum	Selective and progressive atrophy of the cerebellum	-	-	-
Blais et al. [7]	No	Case report	A stroke culminated in the patient’s most recent inflammatory attack. Brain MRI confirmed an acute infarct, without chronic ischemic damage	Acute infarct	-	-	-
Cenedella et al. [8]	No	Case report	Describes the mechanism of cataracts in mevalonic aciduria	-	-	-	-
Bretón Martínez et al. [9]	No	Case report	MRI showed cerebellar atrophy of both hemispheres and vermis	Cerebellar atrophy of both hemispheres and vermis	-	-	-
Dvaladze et al. [10]	Yes	Case report	Ataxia, cerebellar atrophy, and elevated urinary mevalonate, in keeping with mild mevalonic aciduria and associated syndromic retinitis pigmentosa	Cerebellar atrophy	-	-	-
Schwarzer et al. [11]	Yes	Case report	Prenatal ultrasound findings in a fetus with mevalonic aciduria at 28 weeks’ gestation: frontal bossing of the forehead (three-dimensional view)	-	Frontal bossing of the forehead	-	-
Chaudhury et al. [12]	Yes	Case report	White matter changes on MRI consistent with myelination disorder	Extensive parenchymal T2 and FLAIR signal abnormality (defective myelination) and cerebellar atrophy	-	-	-
Hoytema van Konijnenburg et al. [13]	No	Case report	Psychomotor delay and ataxia. MRI showed cerebellar atrophy	Cerebellar atrophy	-	-	-
Chiu et al. [14]	No	Case report	Head MRI showed ependymal cysts along the lateral walls of the lateral ventricles	Ependymal cysts along the lateral walls of the lateral ventricles	-	-	-
Brennenstuhl et al. [15]	Yes	Literature review	11 detailed case reports with neuroimaging findings that included cerebellar atrophy, agenesis of cerebellar vermis, cerebral cortical atrophy, and intracranial cystic lesions	Cerebellar atrophy, agenesis of cerebellar vermis, cerebral cortical atrophy, and intracranial cystic lesions	Multiple periventricular cysts, hyperechoic periventricular white matter, and poor gyration	-	-
Navallas et al. [16]	Yes	Literature review	MRI may demonstrate T2 hyperintensities in the white matter, cerebral atrophy	Non-specific subcortical focus of high signal intensity. Supra- and infratentorial parenchymal volume loss	-	-	-
Torreggiani et al. [17]	No	Case report	A total body MRI was normal except for mild cerebellar hypoplasia and the known interstitial lung disease	Mild cerebellar hypoplasia	-	-	-
Kellner et al. [18]	Yes	Case report	They presented with severe ataxia, pseudophakia due to early-onset cataracts, and progressed retinitis pigmentosa	-	-	Spectral-domain optical coherence tomography of the right eye documented cystoid macular edema and centrally preserved outer retinal layers which markedly decrease paracentrally	-
Lomakina et al. [19]	No	Case report	MA with severe neurological involvement, mimicking Leigh encephalopathy, which is a neurodegenerative disorder associated with dysfunction in mitochondrial energy metabolism	MRI of the brain showed symmetrical T2 hyperintensity of basal ganglia, severe brain atrophy, and thinning of the corpus callosum	-	-	-
Armbrust et al. [20]	Yes	Case report	Orbital tendonitis as a hitherto unreported symptom	Tendonitis with tendomyositis of the inferior rectus and lateral rectus muscles	-	-	-
Prietsch et al. [21]	Yes	Case report	Normal physical and psychomotor development at the time of diagnosis, and MRI scans were normal. During the next years, the patient developed progressive ataxia, and MRI at the age of four years documented the development of severe cerebellar atrophy	Cerebellar atrophy	-	-	-
Mancini et al. [22]	No	Case report	Several clinical signs were present in all three children, including failure to thrive, susceptibility to infections, hepatosplenomegaly, cataracts, and psychomotor disability. Dysmorphic features were more apparent in the two older siblings	-	-	-	Slight dysmorphic features: microcephaly, triangular face, and hypoplastic alae nasi
Ruiz Gomez et al. [23]	Yes	Case report	Patient 1: Brain MRI showed cavities at the frontal and parietal periventricular white matter. Patient 2: MRI revealed progressive cerebellum atrophy. Spectroscopic MRI did not detect evidence of neuroaxonal dysfunction in the central nervous system areas studied. A mild creatine and energetic metabolism deficiency in white matter and cerebellum were detected by abnormal quotient factors. No signs of demyelinization activity were found	Cavities at frontal and parietal periventricular white matter and progressive cerebellum atrophy. Spectroscopic MRI revealed a mild creatine and energetic metabolism deficiency on white matter and cerebellum that were detected by abnormal quotient factors	-	-	-
Pietrasanta et al. [24]	No	Case report	A total body MRI revealed no abnormal mass or bone densities but showed mild cerebellar hypoplasia and confirmed the interstitial lung disease	Mild cerebellar hypoplasia	-	-	-

There appears to be a strong correlation between severe neurological symptoms and neuroimaging findings. For instance, all patients with ataxia showed a cerebellar volumetric loss, while those with structural supratentorial abnormalities presented intellectual disability and failure to thrive [23]. Although a potential relationship between chronic headaches in MA and non-specific punctate white matter signal abnormality has been suggested, there is currently insufficient evidence to establish causation [16].

The novel findings in our case, including polymicrogyria, present a challenge for explanation, as there is no established literature linking polymicrogyria with *MVK* gene mutations. However, there is a well-established association between innate metabolic errors and polymicrogyria, as detailed in the Barkovich et al. classification of malformations, which includes a category for cortical dysgenesis secondary to inborn errors of metabolism [25]. This category encompasses diseases such as mitochondrial and pyruvate metabolic disorders, as well as peroxisomal disorders (e.g., Zellweger syndrome). Additionally, polymicrogyria can result from genetic mutations, it can also occur due to insults such as early infections, hypoxia-ischemia, or trauma. Thus, it is plausible that early onset and severe disease in utero may contribute to this anatomical alteration. However, it remains uncertain whether the findings of polymicrogyria in this case are purely coincidental or have a direct relationship with MA, but these are potential explanations.

The imaging findings suggest structural abnormalities that, in association with symptoms of an autoinflammatory disease, could raise the suspicion of a metabolic disease, which can be confirmed by genetic testing. Furthermore, serial imaging could prove useful in monitoring disease progression, especially in patients where treatments such as IL-1 receptor antagonists are utilized, or in exceptional cases such as the report of dual liver and hematopoietic stem cell transplantation in a patient with atypical MA. There were significant improvements in symptoms and MRI abnormalities in both cases [3,12].

## Conclusions

Neuroimaging findings suggest structural abnormalities that, in association with symptoms of an autoinflammatory disease, could raise the suspicion of a metabolic disease, which can be confirmed by genetic testing. This study highlights both previously documented and novel neuroimaging features. However, it is important to recognize the variability in imaging abnormalities, ranging from dysplastic findings to signal abnormalities.

Additionally, the presence of imaging abnormalities may not be specific to MA alone, as some metabolic and genetic diseases also exhibit similar abnormalities. Therefore, while cases with organic brain abnormalities might tend to be more severe, these findings must be interpreted with caution.

The integration of neuroimaging with clinical and genetic data enhances our understanding of the pathophysiological mechanisms underlying MA. Continued documentation and study of neuroimaging findings will be essential for improving patient diagnosis and management strategies.
